# Rectangular-Normalized Superpixel Entropy Index for Image Quality Assessment

**DOI:** 10.3390/e20120947

**Published:** 2018-12-10

**Authors:** Tao Lu, Jiaming Wang, Huabing Zhou, Junjun Jiang, Jiayi Ma, Zhongyuan Wang

**Affiliations:** 1 Hubei Key Laboratory of Intelligent Robot, School of Computer Science and Engineering, Wuhan Institute of Technology, Wuhan 430073, china; 2 School of Computer Science and Technology, Harbin Institute of Technology, Harbin 150001, China; 3 Electronic Information School, Wuhan University, Wuhan 430072, China; 4 Beijing Advanced Innovation Center for Intelligent Robots and Systems, Beijing Institute of Technology, Beijing 10081, China; 5 School of Computer Science, Wuhan University, Wuhan 430072, China

**Keywords:** image quality assessment, mutual information, superpixel segmentation

## Abstract

Image quality assessment (IQA) is a fundamental problem in image processing that aims to measure the objective quality of a distorted image. Traditional full-reference (FR) IQA methods use fixed-size sliding windows to obtain structure information but ignore the variable spatial configuration information. In order to better measure the multi-scale objects, we propose a novel IQA method, named RSEI, based on the perspective of the variable receptive field and information entropy. First, we find that consistence relationship exists between the information fidelity and human visual of individuals. Thus, we reproduce the human visual system (HVS) to semantically divide the image into multiple patches via rectangular-normalized superpixel segmentation. Then the weights of each image patches are adaptively calculated via their information volume. We verify the effectiveness of RSEI by applying it to data from the TID2008 database and denoise algorithms. Experiments show that RSEI outperforms some state-of-the-art IQA algorithms, including visual information fidelity (VIF) and weighted average deep image quality measure (WaDIQaM).

## 1. Introduction

With the rapid development of digital communication, images are playing increasingly important role in modern society. However, the quality of images is naturally degraded due to image acquisition, compression, storage, and transmission. Image quality assessment (IQA) is a basic problem in the field of image processing research. Image quality determination using only human-in-the-loop-based qualitative measures is time consuming, labor intensive, and cannot be applied to real-time or autonomous systems. Generally, objective IQA metrics can be divided into full-reference (FR), no-reference (NR), and reduced-reference (RR) methods [1,2,3].

A human visual system (HVS) is sensitive to visual quantifiable features such as brightness [4], contrast [5], inter-patch and intra-patch similarities [6], visual saliency [7], fuzzy gradient similarity deviation [8], and frequency content of an image [9]. FR-based IQA metrics are divided into two classes, namely, error statistic-based and HVS-based classes. Error statistic-based methods measure the distance between a distorted image and a reference image at the pixel level, and are less consistent with the HVS. Peak signal-to-noise ratio (PSNR) [10] and mean-squared error (MSE) are the widely used error statistic-based methods. HVS-based methods use visual quantifiable features to construct a visual model. These factors are important in simulating the human perception of image distortion [11,12,13]. A noise quality measure (NQM) [11] uses these factors to find an image quality measure. Wavelet-based visual SNR (VSNR) [12] compares the low-level HVS property of the perceived contrast and the mid-level HVS property of global precedence.

The structural similarity (SSIM) index [14] suggests that the human eye is more sensitive to structural information based on field-of-view, and quantifies the degree of distortion by comparing brightness, contrast, and mechanism similarities. Wang et al. [15] combined the multi-scale SSIM (MS-SSIM) of wavelet domain and obtained improved performance. From the perspective of information theory, Sheikh et al. [16] proposed an information fidelity criterion (IFC) to quantify the mutual information (MI) of reference and distorted images. In [13], IFC was expanded to contain visual information fidelity (VIF). The feature similarity (FSIM) index [17] determines the visual difference of images in the feature domain by comparing the gradient and phase consistency. Li et al. [18] demonstrated the effectiveness of regional MI for IQA. Existing studies [17,19] have shown that VIF and FSIM are more consistent with the subjective results, compared to other traditional algorithms. Recently, Bosse et al. [20] proposed a deep neural networks (the network is based on HVS model) for image quality assessment (WaDIQaM), and achieved state-of-the-art performance.

Distortions, such as noise and blur, are inevitable in non-ideal image degradation and transmission [21,22]. In fact, the scenarios of different IQA applications are also different. Although the abovementioned methods are general, their performances are degraded when the images undergo specific degradations [23,24,25,26]. Our experimental results also prove this point (more details in Section 3.2).

Generally, IQA metrics frequently use fixed-size sliding windows to simulate HVS, such as receptive field [17]. However, they ignore the irregular and inhomogeneous the content and distribution of images, especially the variable spatial configuration information in satellite image. Image segmentation can divide the image into image patches with similar semantics. Existing traditional image segmentation algorithms are mainly divided into three categories, namely, turbopixel/superpixel [27,28] segmentation, watershed segmentation [29,30], active contour [31,32] algorithms. Recent studies conducted in [33,34] show that superpixel [28] provides an state-of-the-art representation of image data.

This study proposes a novel RSEI for IQA. Image patches provided by sliding window ignore the spatial structure information in the image and the correlation between the pixels, and can only measure the quality of the image from the low semantic information level. RSEI utilizes the superpixel segment [28] of the reference image and then clusters the content of the image. The superpixel is used to fully exploit the spatial information to obtain high-level semantic image patches. The distorted image is segmented based on clustering information. Therefore, the weights are automatically generated based on the IE of the reference image patch. RSEI uses MI to describe the changes between image patches.

Overall, the contributions of this paper are highlighted as follows:

1. The proposed IQA metric semantically divides the image into multiple flexible patches based on superpixel to accurately measure multi-scale objects in images. Here, the superpixel of images provides the variable spatial configuration information.
2. A weighting scheme that determines the importance of an image patch based on its information volume is introduced. This weighting scheme reveals the attention-seeking mechanism of HVS.
3. The proposed IQA metric focuses on the inevitable problems of image degradation and compression.

The remainder of this paper is structured as follows. In Section 2, we describe the framework of the proposed method. Experiment signals is analyzed in Section 3. In addition, comparison is performed among the proposed metric and some representative IQA methods to show the superiority of the proposed method. Discussion and conclusion are summarized in Section 4 and Section 5, respectively.

## 2. Rectangular-Normalized Superpixel Entropy Index

MI measures the degree of image distortion by quantifying the information dependence between reference image *Y* and distorted image $\hat{Y}$ [35,36]. The joint entropy $H(\hat{Y},Y)$ of images $\hat{Y}$ and *Y* is defined as follow:

(1) $$\;H\left(\hat{Y},Y\right)=\sum_{s\in \hat{Y}}\sum_{t\in Y}p(s,t)\log p\left(s,t\right).$$

where *s* and *t* represent the gray-scale value of the image, and p(s,t) is the joint probability of *s* and *t*. Thus, MI is defined as follow:

(2) $$\;\begin{array}{c} I\left(\hat{Y},Y\right)=H\left(\hat{Y}\right)+H\left(Y\right)-H\left(\hat{Y},Y\right)\\ =\sum_{s\in \hat{Y}}\sum_{t\in Y}p\left(s,t\right)\log\frac{p(s,t)}{p(s)p(t)}, \end{array}$$

where $H(\hat{Y})$ and H(Y) are the entropies, p(s) represents the probablity distribution.

The greater the MI between the images, the greater the similarity information between images will be. However, MI ignores the visual perception characteristics of HVS, such as image patch weighting and contrast sensitivity. For an intuitive comparison, MI is normalized as follows [37]:

(3) $$\;NMI\left(\hat{Y},Y\right)=\frac{2I(\hat{Y},Y)}{H(\hat{Y})+H\left(Y\right)}.$$

IE reflects the amount of information in an image. However, the interference caused by distorted images increases the amount of information in that image, and this additional information causes a negative impact. Therefore, MI is not robust to the interference in the image, resulting in an inaccurate evaluation of image quality of the distorted image.

Various semantic image patches are important to the overall image. Different semantic objectives in the image have different levels of importance to the image. For objects with small variation, the amount of information, such as the sky and sea, and distortion have a small effect on the subjective quality of the image. For larger patches of information, such as airplanes and ships, each image section contains considerable gradients and structural information [38].

**Figure 1:**
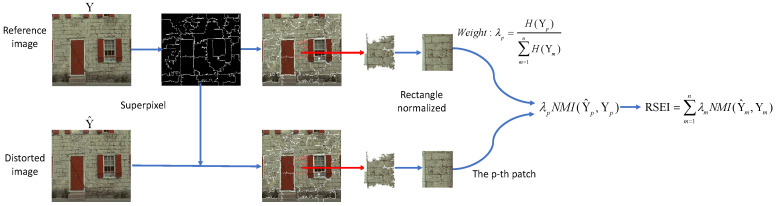
Illustration of the RSEI. Superpixel segmentation provides clustering information of spatial pixels which contains flexible image semantic information.

RSEI is illustrated in Figure 1. Reference image *Y* is divided into *n* image patches by semantic segmentation [28]. The corresponding segmentation label is recorded as $l_{Y}$. The resulting distorted image $\hat{Y}$ is divided by label $l_{Y}$.

The shape of the image patch after semantic segmentation is irregular. In order to be able to do calculations, it is usually filled as a rectangle. Padding areas of 0 or 255 [39] are treated as exactly the same area, whether pixel-based or HVS-based, which will add additional error terms. Therefore, we use the minimum area of bounding rectangle-normalized to normalize the image patch by self-padding to avoid excessive filling that affects segmentation. The convex hull of the image patch is recorded as ${\left\{hx_{i},hy_{i}\right\}}_{i=1}^{t}$, where *t* is the number of convex hull points. Minimum area *S* is the rectangle defined as:

(4) $$\;\theta =\mathrm{unique}\left(\;\mathrm{mod}\;\left(\arctan\left(\frac{hy_{i+1}-hy_{i}}{hx_{i+1}-hx_{i}}\right),\frac{\pi }{2}\right)\right),$$

(5) $$\;\begin{array}{c} S=arg\min_{\theta }\left\{\left(\max\left(\left[hx,hy\right]*\left[\begin{array}{c} \sin(-\theta )\\ \cos(-\theta ) \end{array}\right]\right)-\right.\right.\\ \left.\min\left(\left[hx,hy\right]*\left[\begin{array}{c} \sin(-\theta )\\ \cos(-\theta ) \end{array}\right]\right)\right)*\\ \left(\max\left(\left[hx,hy\right]*\left[\begin{array}{c} cos(-\theta )\\ -sin(-\theta ) \end{array}\right]\right)-\right.\\ \left.\left.\min\left(\left[hx,hy\right]*\left[\begin{array}{c} cos(-\theta )\\ -sin(-\theta ) \end{array}\right]\right)\right)\right\}, \end{array}$$

where unique is used to obtain the non repetitive element, mod is the modulo function. θ is the angle set of bounding rectangle that is moved into the first quadrant.

**Figure 2:**
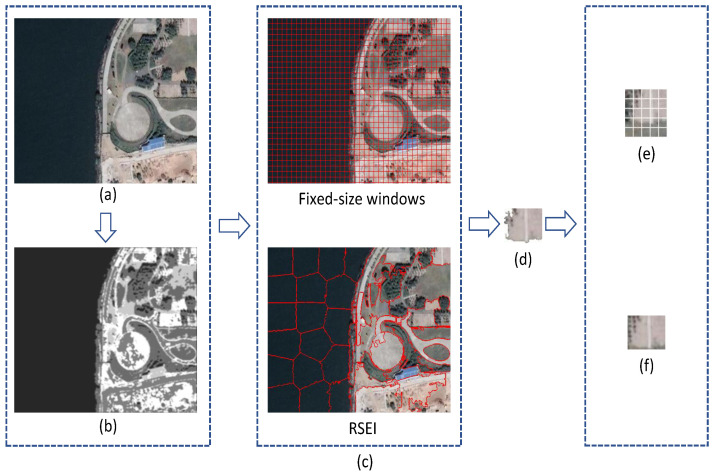
Differences between RSEI and the fixed-size sliding window. (**a**) Reference image. (**b**) Saliency map of the reference image. (**c**) Segmentation results. (**d**) Image patch in the upper-right corner of semantic segmentation map. (**e**) Completely overlay the image patch (**d**) by fixed-size windows. (**f**) Completely overlay the image patch (**d**) by RSEI.

Figure 2 shows the differences between the fixed-size sliding window and the proposed method. Image saliency [40] is an important visual feature in an image, that emphasizes the degree of importance of a region for human eye perception. The segmentation results of RSEI conform to the saliency map of the image. RSEI covers an irregular semantic patch 3(d) with the smallest rectangle, and the sliding windows ignore the semantics of the image patch and cover many unrelated areas.

Then, the weight of the image patch is determined based on its IE. The larger the amount of information in the image is, the more important each object information will be to the overall assessment of the image. Patches with a small amount of information should occupy a small proportion. The weight of the p-th patch is defined as follows:

(6) $$\;\lambda_{p}=\frac{H(Y_{p})}{\sum_{m=1}^{n}H(Y_{m})},$$

where *n* represents the total number of segmented image patches and $Y_{p}$ denotes the *p*-th image patch. RSEI is defined as follows:

(7) $$\;RSEI=\sum_{m=1}^{n}\lambda_{m}NMI\left({\hat{Y}}_{m},Y_{m}\right)$$

Our proposed RSEI is described in Algorithm 1. The code has been made publicly available at https://github.com/jiaming-wang/RSEI.

**Algorithm 1** RSEI index for IQA**Input:** Initialize the following parameters. (1) Reference images *Y*; (2) distorted images $\hat{Y}$; (3) number of image patches *n*; **Superpixel:** (4) Superpixel segmentation is performed on the reference image *Y*. The corresponding segmentation label is recorded as $l_{Y}$; (5) Normalizing reference images *Y* by label $l_{Y}$; (6) Minimum area bounding rectangular-normalized by using Formula (5). The divided image patches are normalized to ${\left\{Y_{i}\right\}}_{i=1}^{n}$, the distorted images $\hat{Y}$ are normalized to ${\left\{{\hat{Y}}_{i}\right\}}_{i=1}^{n}$. **RSEI:** **for each patch**
i=1 to *n* (7) Compute weighted $\lambda_{i}$ by Formula (6); (8) Compute MI $NMI({\hat{Y}}_{i},Y_{i})$ by Formula (3); **end** (9) Compute RSEI by Formula (7).

## 3. Experiments

### 3.1. Databases

The TID2008 dataset [41] is a commonly used public database in the IQA community. The dataset contains 25 reference images and 1,700 distorted images. Each reference image corresponds to 68 different distorted images and includes 17 types of distortion. The MOS of the images is scored by 838 observers. The image size is 512×384 pixels. All images are RGB images. However, all IQA algorithms are used in single-channel images. We convert the image pixels to YCbCr color space by using only the Y channel for testing.

Four common performance metrics are used to evaluate the performance of assessment methods. The Kendall rank-order correlation coefficient (KROCC) and Spearman rank-order correlation coefficient (SROCC) can be effectively used to measure the prediction monotonicity of an IQA metric. The two other metrics are the Pearson linear correlation coefficient (PLCC) and root MSE (RMSE) between MOS and objective scores after nonlinear regression. An excellent method indicates high KROCC, SROCC, and PLCC while low RMSE score [42].

### 3.2. Limitations of Existing IQA Algorithms

**Figure 3:**
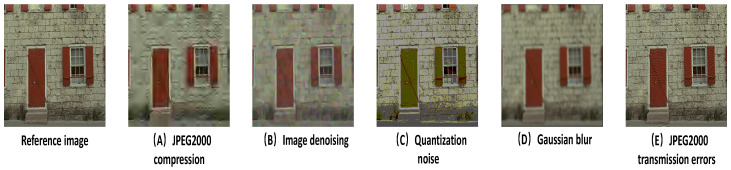
(**A**–**E**) are the distorted versions of a reference image in TID2008 database. The distortion types of (**A**–**E**) are JPEG2000 compression, image denoising, quantization noise, Gaussian blur, and JPEG2000 transmission errors, respectively.

Figure 3, shows five types of distortion, namely, JPEG2000 compression, image denoising, quantization noise, Gaussian blur, and JPEG2000 transmission errors, all of which are selected from TID2008 [41]. A shown in Table 1, the last column is the mean opinion scores (MOS) of the images, and the first five columns are the results of PSNR, SSIM, VIF, FSIM, and rectangle-normalized superpixel entropy index (RSEI), respectively. Neither the state-of-the-art deep learning-based algorithm WaDIQaM nor the best performing VIF and FSIM can accurately describe these distortion changes.

### 3.3. Parameter Settings

**Figure 4:**
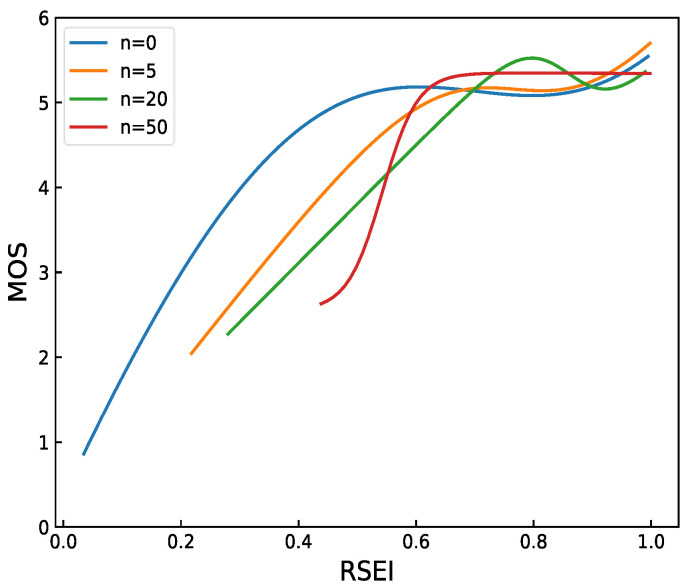
Fitting curve of number *n* of different image patches. RSEI has better performance when the values of *n* are 20 and 50.

The number of image patches is an important parameter used in RSEI. The first six reference images are selected from the TID2008 [41] reference image and the corresponding 408 distorted images for parameter tuning. We set *n* at 0, 5, 20, and 50 and then perform RSEI evaluation. The curve fitting results are shown in Figure 4. The patch is inaccurate when *n* is 0 and 5. RSEI cannot accurately evaluate all images with high MOS, and performance is not constantly improved with the increase of *n*. The image patch increases when *n* is 50, which leads to the low recognition degree of RSEI for MOS greater than 5 and high time complexity. Thus, we set *n* to 20.

However, there is no MOS score in the actual scenario, so that the optimal solution of *n* cannot be directly obtained. The larger the value of *n*, the finer the classification, and the longer it will take. Therefore, the value of *n* depends on the accuracy requirement of the task in the actual scene.

### 3.4. Performance Comparison with State-of-the-Art IQAs

The evaluation results are compared with some representative FR IQA metrics, including some state-of-art algorithms:

- SSIM [14]—a widely used methods for IQA
- MS-SSIM [15]—the multi-scale SSIM (MS-SSIM) of wavelet domain
- FSIM [17]—the best-performance IQA method based on structural information
- VSNR [12]—wavelet-based visual SNR
- IFC [16]—an information fidelitycriterion using natural scene statistics
- NQM [11]—a noise quality measure
- VIF [13]—the excellent image quality assessment method based on information theory
- WaDIQaM [20]—the state-of-the-art image quality assessment method based on deep learning

Here, the wavelet domain version of VIF is used. Other comparison algorithm results are provided by TID2008 [41] datasets, except for FSIM and VIF. For FSIM and WaDIQaM, we directly use the open-source codes provided by the author and the parameters in this study. The experiment uses 25 reference images and the corresponding six types of distorted images, which are JPEG2000 compression, JPEG compression, image denoising, quantization noise, Gaussian blur, and JPEG2000 transmission errors.

**Figure 5:**
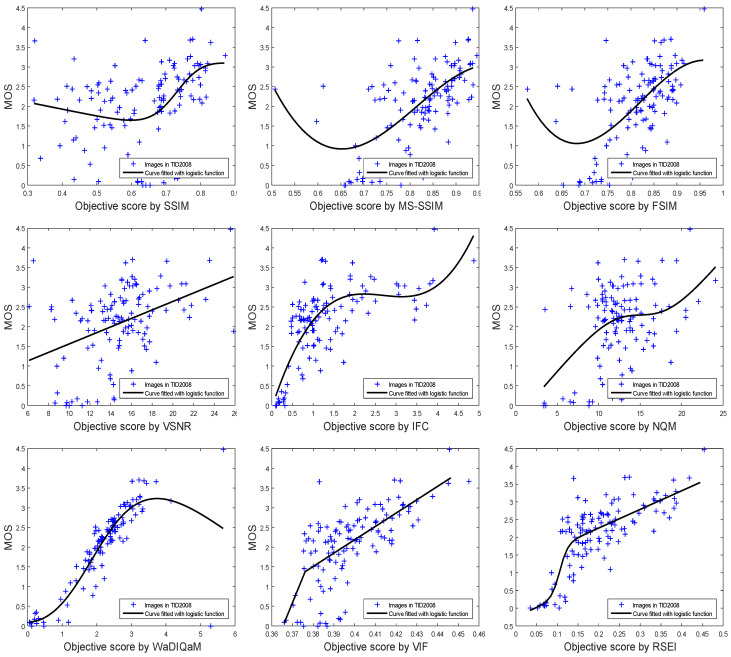
Scatter plots of subjective MOS versus scores obtained by model prediction.

The curve fitting to MOS and image objective scoring is shown in Figure 5. All the scores of the IQA metrics are listed in Table 2. RSEI obtains the best objective score for SROCC, PLCC, and RMSE, and the next best score for KROCC. For the abovementioned distortion types, the performance of RSEI correlates more consistently with the subjective evaluations than do the other methods.

### 3.5. Running Time

**Figure 6:**
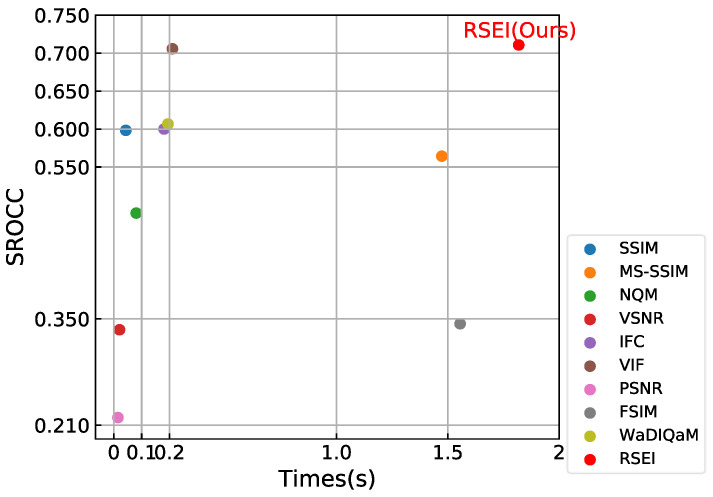
Mean running time (seconds) of all 150 samples for different algorithms.

The running time comparisons of all algorithms are shown in Figure 6. WaDIQaM has a shorter test time, however it takes a lot of time for data training, which could not be ignored. RSEI remembers the semantic segmentation labels, so it does not increase dramatically when testing large amounts of data. Combining the data in Table 2, it is clear that RSEI has the best performance by sacrificing a little time. We implement all algorithms in the experiments under the same hardware configuration, which are as follows: Intel Core i5-6300HQ CPU @2.30 GHz, 8 GB RAM, and NVIDIA GeForce GTX 950M.

### 3.6. Application of Denoise Algorithmic Scenario

Although existing IQA metrics are designed to measure standard datasets, they do not evaluate images that undergo a complex nonlinear transformation in a real algorithmic scenario. Since there is no MOS score in this algorithmic scenario, the deep learning algorithm is difficult to apply [43,44].

**Figure 7:**
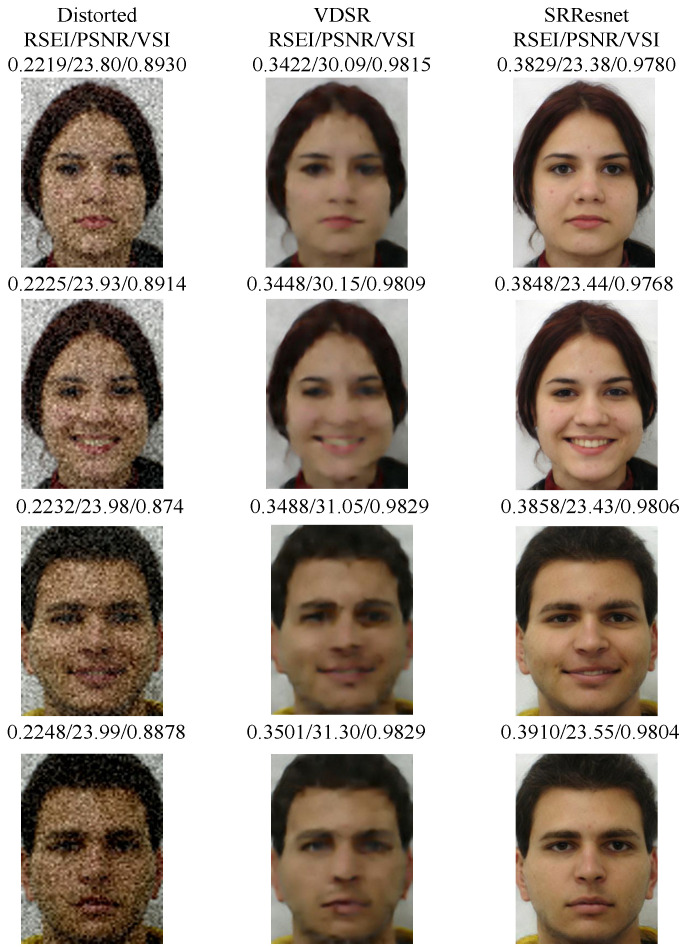
Inconsistency between PSNR/VSI values and perceptual quality. The images are distorted image with noise level σ=20, VDSR, and SRResnet. The result of SRResnet has the highest perceptual quality, where its PSNR/VSI values are low and RSEI value is high.

The FEI faces database [45] includes 400 images of 200 people (100 men and 100 women). Each person has two frontally aligned face images, where the first one is a frontal facial image and second one is a smiling image. The image size is 260×360 pixels. Figure 7 shows several denoising results on the FEI face dataset [45] in which the images are added with different levels of Gaussian noise. σ is the standard deviation of the added noise to the images. The first column is the distorted image used as the input noise image with σ=20. The last two columns are the denoising results of VDSR [46] and SRResnet [47], respectively. Clearly, SRResnet has a stronger denoising effect, the results also show more fluent details and color deviations.

The result of SRResnet has the highest perceptual quality, where its PSNR/VSI [7] values are low and its RSEI value is high. The performance of RSEI consistently correlates with subjective evaluations. PSNR focuses on pixel-level differences and cannot measure the image quality in HVS. VSI effectively shows that VDSR is similar in visual saliency to the ground truth but is insensitive to the detailed information of the image. However, RSEI measures the information fidelity in semantic structure patches, combines the advantages of two methods, and obtains accurate evaluation results.

## 4. Discussion

### 4.1. Traditional Methods

Traditional algorithms use fixed-size sliding windows to simulate receptive fields, which does not incur additional running time. They ignore the spatial structure information in the image and the correlation between the pixels, and can only measure the quality of the image from the low semantic information level. RSEI semantically segments images for flexible processing of image patches, that benefited from the contributions of superpixel. However, it also leads to an increase in the running time of the algorithm.

### 4.2. Deep Learning-Based Methods

Deep learning-based methods rely on the priori information provided by a large number of training datasets to obtain the excellent performance. Since there is less public dataset for IQA, complex preprocessing is required to obtain data augmentation [20]. Meantime, the network training process depends on the acceleration of hardware devices (GPU) which is the limitation of WaDIQaM. The distortion in the real application scenario is more complex, and it is difficult to ensure the deep learning-based algorithm with good generalization ability.

## 5. Conclusions

This study introduced a novel RSEI for QIA. Semantic segmentation was applied to the variable spatial configuration information rather than using a fixed-size sliding window. RSEI is based on the perspective of a variable receptive field and IE to better measure multi-scale objects in satellite images. RSEI assigns weights to the image patch based on its degree of information richness. We verified the effectiveness of RSEI by applying it to the data from the TID2008 database, denoising algorithms, and inaccurate supervision application scenarios. We believed that the proposed approach is appropriate to this satellite application scenario, in which the ground truth of satellite images is frequently unavailable.

## Figures and Tables

**Table 1:** Subjective scores with different type of distortion. From this table, traditional image quality measures such as PSNR, SSIM, VIF and FSIM are not always complied with distribution of MOS scores.

Type of Distortion	PSNR	SSIM	VIF	FSIM	WaDIQaM	RSEI	MOS
**JPEG2000 compression**	21.0628	0.3939	0.0858	0.7472	1.9727	0.2524	1.0000
**Image denoising**	21.0453	0.4397	0.1305	0.7688	2.2150	0.2605	1.2000
**Quantization noise**	21.1297	0.7806	0.3330	0.8907	2.4559	0.2592	2.1667
**Gaussian blur**	21.0833	0.3859	0.1125	0.7422	2.4214	0.2640	2.1765
**JPEG2000 transmission errors**	20.7817	0.7106	0.3617	0.9112	4.1493	0.3100	3.1765

**Table 2:** Performance comparison of IQA metrics. Red color indicates the best performance and blue color indicates the second best performance.

Category	Assessment Methods	KROCC	SROCC	PLCC	RMSE
**Error statistic-based**	**PSNR**	0.1328	0.2201	0.2876	0.9183
**HVS-based**	**SSIM**	0.4095	0.5983	0.7521	0.6669
	**MS-SSIM**	0.4476	0.5643	0.8200	0.5737
	**NQM**	0.3333	0.4893	0.7106	0.7053
	**VSNR**	0.2881	0.3357	0.8561	0.5131
	**IFC**	0.4286	0.6000	0.7279	0.6873
	**VIF**	0.6000	0.7057	0.8578	0.5152
	**FSIM**	0.2571	0.3436	0.6532	0.7590
	**WaDIQaM**	0.6067	0.7067	0.8592	0.4911
	**RSEI**	0.5619	0.7107	0.8593	0.5128
